# The Fire and Smoke Model Evaluation Experiment—A Plan for Integrated, Large Fire–Atmosphere Field Campaigns

**DOI:** 10.3390/atmos10020066

**Published:** 2019

**Authors:** Susan Prichard, N. Sim Larkin, Roger Ottmar, Nancy H.F. French, Kirk Baker, Tim Brown, Craig Clements, Matt Dickinson, Andrew Hudak, Adam Kochanski, Rod Linn, Yongqiang Liu, Brian Potter, William Mell, Danielle Tanzer, Shawn Urbanski, Adam Watts

**Affiliations:** 1 University of Washington School of Environmental and Forest Sciences, Box 352100, Seattle, WA 98195-2100; 2 US Forest Service Pacific Northwest Research Station, Pacific Wildland Fire Sciences Laboratory, Suite 201, Seattle, WA 98103, USA; 3 Michigan Technological University, 3600 Green Court, Suite 100, Ann Arbor, MI 48105, USA; 4 US Environmental Protection Agency, 109 T.W. Alexander Drive, Durham, NC 27709, USA; 5 Desert Research Institute, 2215 Raggio Parkway, Reno, NV 89512, USA; 6 San José State University Department of Meteorology and Climate Science, One Washington Square, San Jose, CA 95192-0104, USA; 7 US Forest Service Northern Research Station, 359 Main Rd., Delaware, OH 43015, USA; 8 US Forest Service Rocky Mountain Research Station Moscow Forestry Sciences Laboratory, 1221 S Main St., Moscow, ID 83843, USA; 9 Department of Atmospheric Sciences, University of Utah, 135 S 1460 East, Salt Lake City, UT 84112-0110, USA; 10 Los Alamos National Laboratory, P.O. Box 1663, Los Alamos, NM 87545, USA; 11 US Forest Service Southern Research Station, 320 Green St., Athens, GA 30602-2044, USA; 12 US Forest Service Missoula Fire Sciences Laboratory, 5775 US Highway 10 W Missoula, MT 59808-9361, USA

**Keywords:** mixed conifer forest, southern pine forest, wildland smoke, fire behavior, plume dynamics, dispersion, smoke chemistry

## Abstract

The Fire and Smoke Model Evaluation Experiment (FASMEE) is designed to collect integrated observations from large wildland fires and provide evaluation datasets for new models and operational systems. Wildland fire, smoke dispersion, and atmospheric chemistry models have become more sophisticated, and next-generation operational models will require evaluation datasets that are coordinated and comprehensive for their evaluation and advancement. Integrated measurements are required, including ground-based observations of fuels and fire behavior, estimates of fire-emitted heat and emissions fluxes, and observations of near-source micrometeorology, plume properties, smoke dispersion, and atmospheric chemistry. To address these requirements the FASMEE campaign design includes a study plan to guide the suite of required measurements in forested sites representative of many prescribed burning programs in the southeastern United States and increasingly common high-intensity fires in the western United States. Here we provide an overview of the proposed experiment and recommendations for key measurements. The FASMEE study provides a template for additional large-scale experimental campaigns to advance fire science and operational fire and smoke models.

## 1. Introduction

Fire and smoke models are essential tools for wildland fire decision-making and planning. However, many models that currently drive operational systems are used without adequate validation and evaluation because of the lack of suitable data. As a consequence, the limits of applicability and expected errors are not defined, and results may not be realistic under certain conditions [1,2]. Accurate estimates of fire and smoke emissions and dispersion from wildland fires are highly dependent on the characterization of many interrelated variables, including area burned, pre-burn biomass of fuel bed components and conditions, fuel consumption by combustion phase, fire behavior, heat release, plume dynamics, meteorology, and smoke chemistry. Reliable predictions of smoke production and dispersion are fundamentally based on modeling fire–atmosphere interactions, including wildland fire behavior and plume dynamics.

Complex physics-based smoke models include, to different approximations, fire and atmosphere dynamics that drive buoyancy-induced plume rise and smoke transport. Currently fire weather forecast coupled models such as the Weather Research Forecasting – Spread FIRE [3,4], MesoNH-ForeFire [5], and Coupled Atmosphere Wildland Fire-Environment models [6,7] utilize simplified fire spread models, and localized smoke models such as Daysmoke [8] approximate the sources of heat and mass that generate the buoyant plume and smoke (Table 1). These models resolve plume dynamics but parametrize combustion-related processes to enable faster than real time simulations of landscape scale (thousands of ha) wildland fires at spatial resolutions of hundreds of meters. In contrast, physics-based models such as FIRETEC [9] and the Wildland-urban-interface Fire Dynamics Simulator [10] explicitly account for the processes of gas-phase combustion and vegetation consumption in addition to plume rise and smoke generation. Computational fluid dynamics models of fire–atmosphere interactions require relatively high-resolution computational three-dimensional grid cells (e.g., 1–5 m^3^), and the resulting high computational demand precludes their routine use on large domains (e.g., greater than approximately 10 ha).

The Fire and Smoke Model Evaluation Experiment (FASMEE) is a proposed observational campaign to evaluate current operational fire and smoke modeling systems and support the advancement of newer models and systems into operational use. Integrated, large research campaigns are critical for making advances in wildland fire behavior, fire effects, and smoke science. An investment in coordinated sampling of fire–atmosphere interactions not only benefits fire and smoke science but also ultimately benefits fire and fuels managers, who rely on the best-available operational fire and smoke models to guide wildland fire management.

The need for integrated, cross-discipline observations to support operational fire and smoke modeling has been highlighted in recent synthesis reports, including the Joint Fire Science Program (JFSP) Smoke Science Plan [12], the Smoke and Emissions Model Intercomparison Project (SEMIP) [13], the Fire and Smoke Model Evaluation workshop and report [14], a special session on Wildland Fire Behavior and Smoke [15], the Prescribed Fire Combustion Atmospheric Dynamics Research Experiment (RxCADRE) special issue [16], and the joint National Aeronautics and Space Administration (NASA) and National Oceanic and Atmospheric Administration (NOAA) Fire Influence on Regional to Global Environments and Air Quality (FIREX-AQ) white paper [17]. Successful collaborations in past field campaigns, including RxCADRE and the Department of Defense’s (DOD) Strategic Environmental Research and Development Program (SERDP)-funded fine-scale combustion studies, led to the JFSP partnering with the Department of Defense’s Environmental Security Technology Certification Program (ESTCP) to initiate the FASMEE planning phase (Phase 1).

The FASMEE study plan is guided by the overarching question, “*How do fuels, fire behavior, fire energy, and meteorology combine spatially to determine the development and dynamics of near-source plumes and the long-range transport of smoke and its chemical evolution?*” To address this question, observational measurements of coupled fire–atmosphere dynamics are needed that are integrated over space and time to quantify a complex set of physical phenomena. To meet objectives that define a successful experiment, cross-scale measurements are necessary to adequately resolve buoyant emissions as well as plume development and smoke dispersion from sufficiently large fire events.

**Figure 1.**
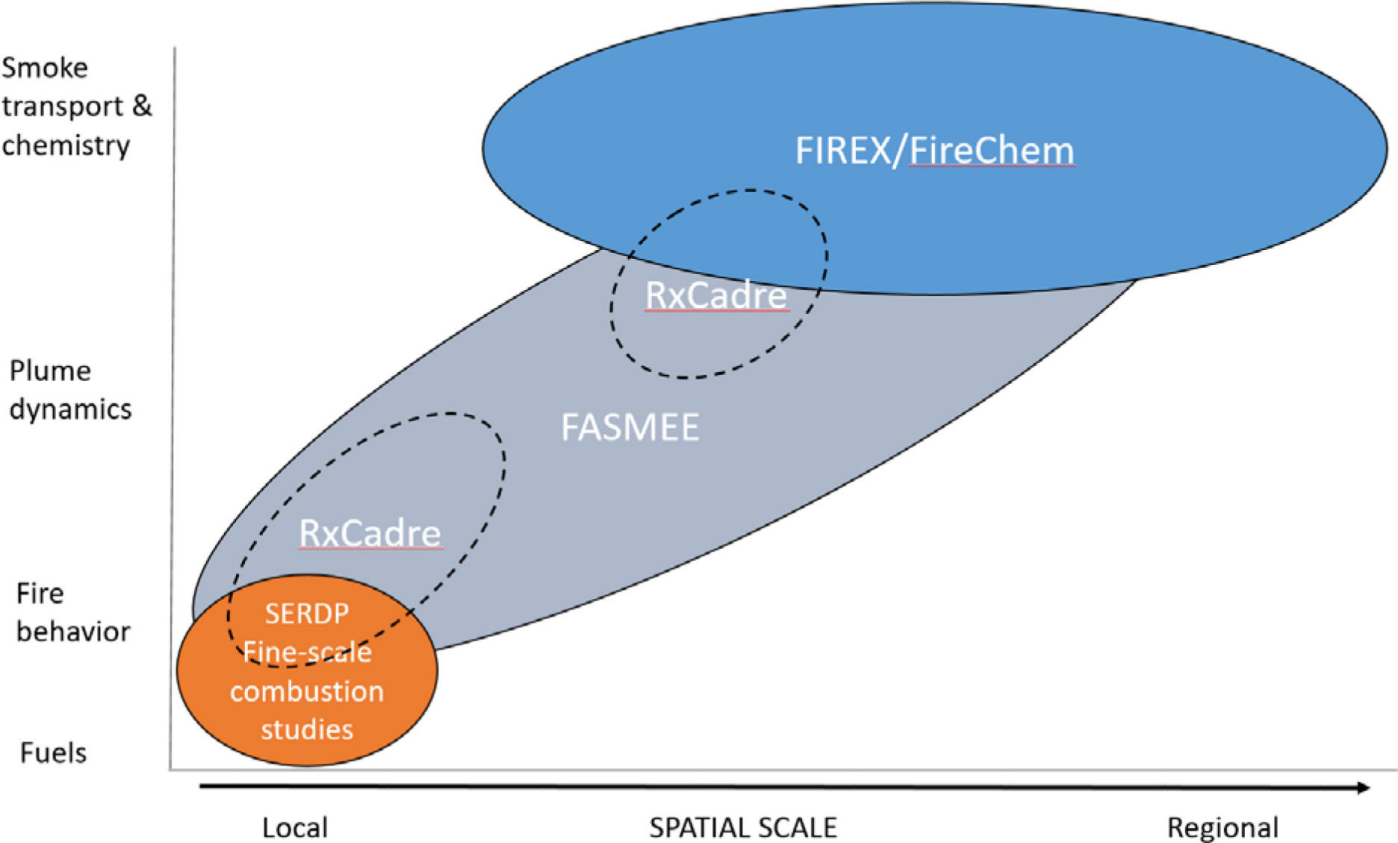
Conceptual diagram showing the spatial scale (x-axis) and discipline focus (y-axis) of proposed fire and smoke field campaigns.

FASMEE is conceived as part of a coordinated set of field campaigns on wildland fire and smoke, spanning a range of spatial scales (Figure 1). At the broadest scale, plume sampling campaigns will evaluate smoke chemistry from large wildland fire events, including the joint NOAA and NASA Fire Influence on Regional and Global Environments Experiment and wildland fire chemistry experiment (FIREX-AQ, summer 2019). At the finest scale, a series of SERDP-funded projects are being conducted in laboratories and as small field experiments to improve the understanding of fine-scale wildland fire combustion processes. The FASMEE campaign addresses meso-scale fire–atmosphere dynamics, starting from ground-based fuel characterization to observation of fire heat release, local meteorology and plume dynamics, and ending with smoke dispersion and chemistry measurements.

**Figure 2.**
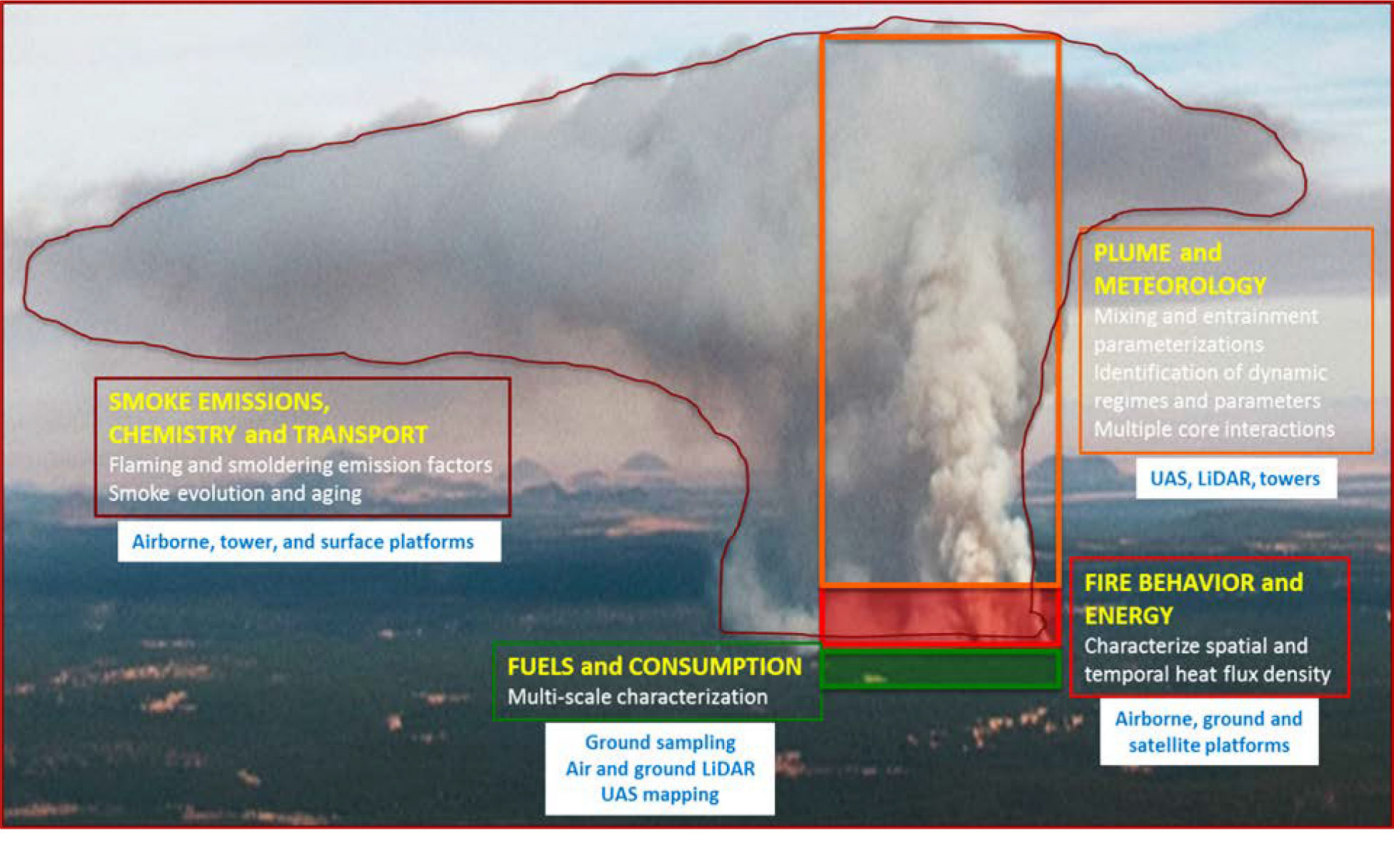
Spatial representation of the four Fire and Smoke Model Evaluation Experiment disciplines and applicable measurement platforms: (1) Fuels and consumption, (2) fire behavior and energy, (3) meteorology and plume dynamics, and (4) smoke emissions and chemistry.

Among these campaigns, FASMEE provides a comprehensive, cross-scale perspective in that it specifically addresses the fuels, fire, and plume development that lead to smoke emissions and injection into the broader atmospheric circulation, as well as near-fire smoke chemistry and plume aging (Figure 2). Developing geospatially and temporally integrated observational datasets that span source characterization, fire behavior, plume development, and smoke dispersion will require multiple experiments across different ecosystems and require substantial investment by international collaborators.

An additional aspect of FASMEE stems from extensive experimental planning performed by a team of scientists, covering all the main FASMEE scientific areas. With the active involvement of fire and smoke modelers, the measurement priorities and specifications have been defined in the context of particular modeling challenges and needs. Pre-burn weather analyses and numerical simulations performed during the planning stage of FASMEE delivers information on the desired burn conditions [18], defines statistically typical burn days, and provides guidelines on measurement strategies and optimal sensor placement [19].

**Figure 3.**
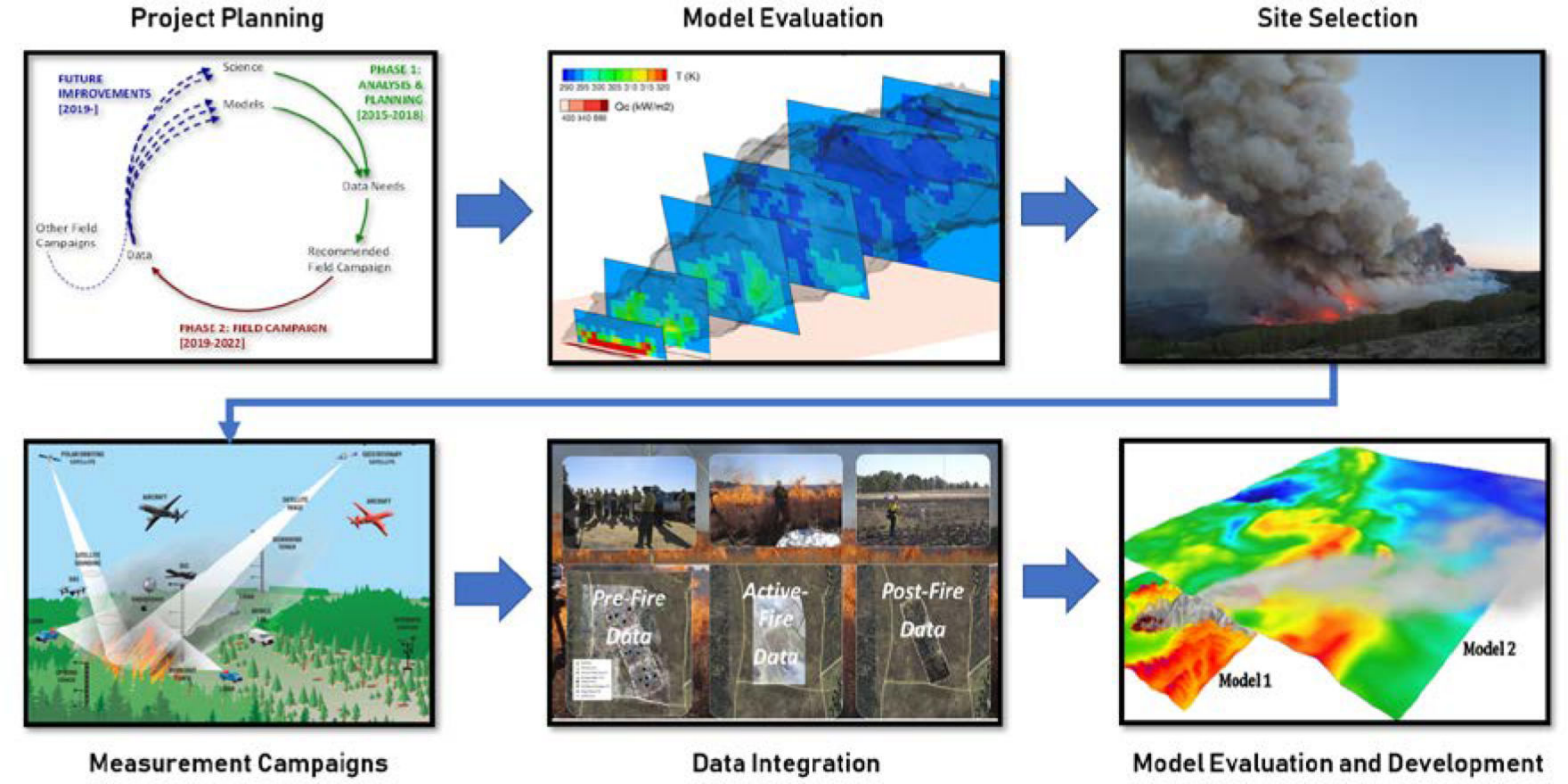
Project flow diagram from the planning stages of the Fire and Smoke Model Evaluation Experiment, including the evaluation of existing models and past campaigns, site selection for large prescribed burn opportunities, spatially and temporally coordinated measurement campaigns, compilation of geospatial datasets, and application to model evaluation and development.

This paper reviews the motivation for integrated measurement campaigns, an overview of FASMEE measurement objectives by discipline, and the discussion of how large fire campaigns can inform model evaluation and development for both scientific understanding and operational application. The FASMEE study plan, abridged here, provides a template for additional large-scale campaigns that together can advance fire science and operational fire and smoke models [20]. A work flow of FASMEE from the planning phase, measurement campaigns and application to model evaluation and development is presented in Figure 3.

## 2. Approach—Field Campaigns

The FASMEE campaign will target large fires with the potential for substantial plume development. An extensive search for field campaign options was undertaken to identify opportunities, including sites across the United States and Canada. In the initial phase of FASMEE, three measurement campaigns were identified based on their ability to meet identified needs and requirements for burns and measurements, host agency partners, cost estimates, and the range of anticipated fire behavior (Table 2).

With JFSP and USDA Forest Service support, the FASMEE planning team collaborated with the 2018 NSF Western Wildfire Experiment for Cloud Chemistry, Aerosol Absorption and Nitrogen (WE-CAN) and will participate in the 2019 Joint NASA and NOAA Fire Influence on Regional to Global Environments and Air Quality (FIREX-AQ) campaigns. Coordination with these campaigns offers a rare opportunity to link aircraft smoke and satellite fire measurements with the characterization of wildfire and plume dynamics. These large aircraft campaigns, coordinated with FASMEE, will also provide a better understanding of the dynamics controlling near-source plumes and the resulting chemical composition, aging, and transport of smoke through measurements and observations of fuel, fire behavior, fire energy, and meteorology.

**Figure 4:**
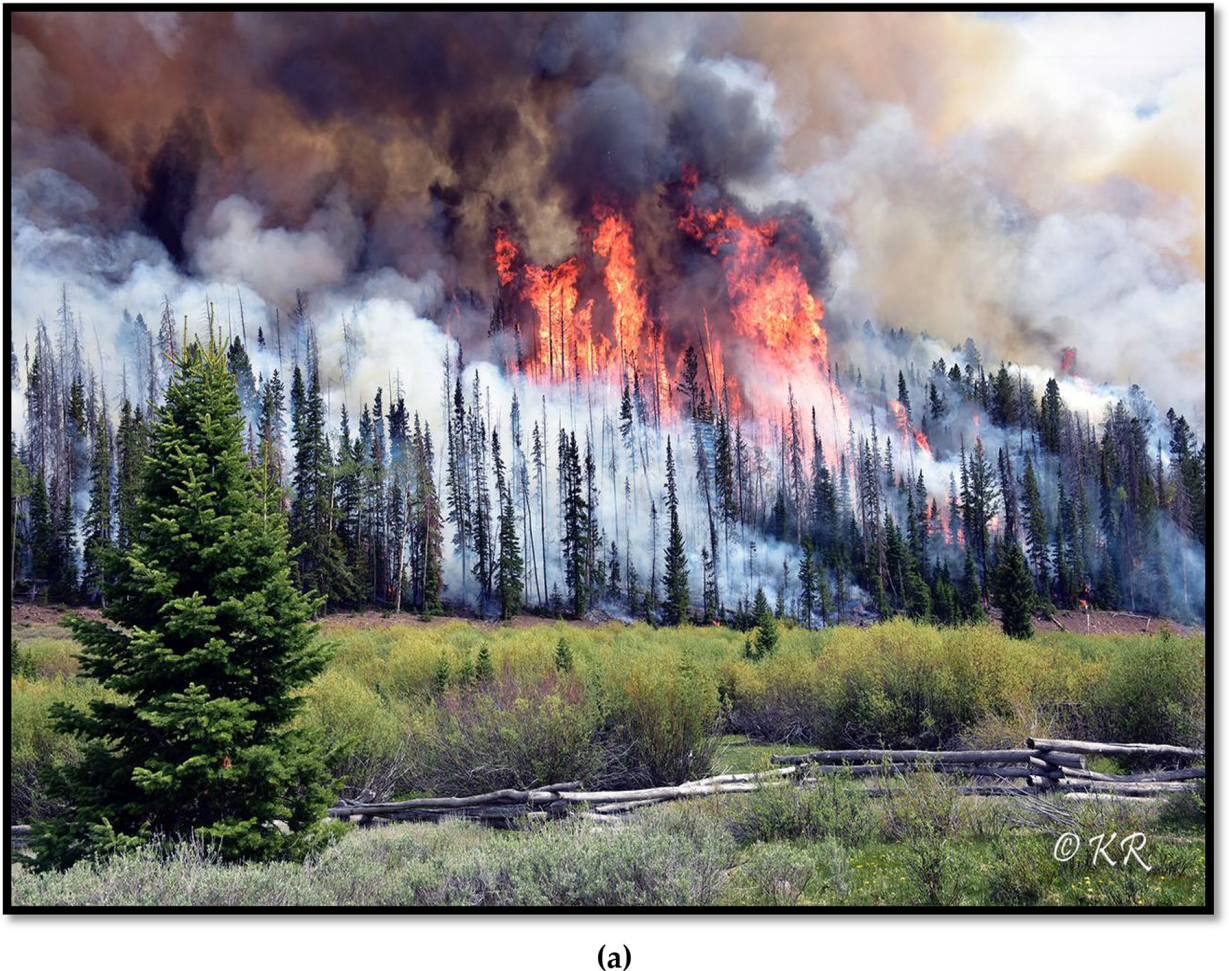

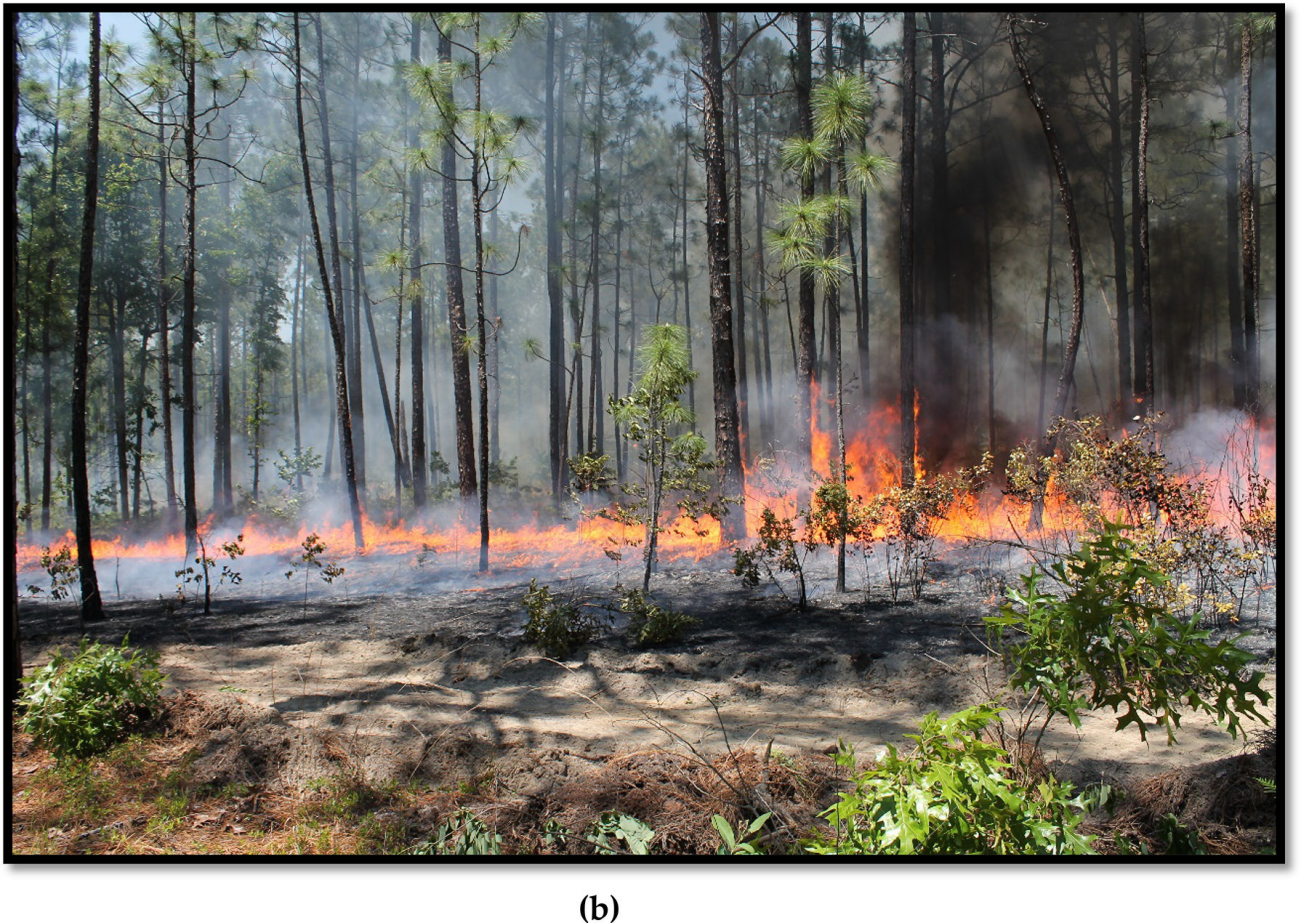
**(a).** Prescribed stand-replacement burn at Fish Lake, Utah (photo credit: Kreig Rasmussen). **(b)**. Understory burn at Fort Jackson, South Carolina (photo credit: Roger Ottmar).

The Southwestern FASMEE Campaign is intended to target prescribed, stand-replacement burns completed under heavy surface fuel loads (50–100 Mg ha^−1^) and high intensity surface and crown fires with dynamic long-range plumes relevant for smoke management. The potential to conduct measurements from prescribed crown fires presents a rare opportunity to sample the spectrum of fuel, fire behavior, and plume dynamics in a wildland fire that resembles a stand-replacing wildfire. Candidate host sites for the Southwestern Campaign have been identified as Fishlake National Forest (NF) in Utah and the North Kaibab Ranger District (RD) of the North Kaibab National Forest in Arizona due to their high fuel loadings, large burn units, and histories of prescribed burning (Figure 4a). Fishlake NF has initiated a large burn campaign to reestablish the quaking aspen (*Populus tremuloides*) stands in the mixed conifer forests using planned stand-replacement fires. Fishlake NF has been recently surveyed with aerial LiDAR, providing full coverage that FASMEE can use to enhance the characterization of canopy fuels. Fuels for all sites are mixed conifer and aspen forests with insect and disease damage following decades of fire exclusion. North Kaibab RD has an ambitious fuels management program and has established hundreds of permanent forest inventory and fuel plots throughout both ponderosa pine (*Pinus ponderosa*) and mixed conifer forests, including existing LiDAR coverage for much of the forest area. The district regularly conducts large (>500 ha) underburns of ponderosa pine and mixed conifer forests. The forest inventory and fuel data are available to FASMEE and will supplement the data collection planned for improving the representation of the fuels.

Fort Stewart in Georgia and the Department of Energy’s (DOE) Savannah River Site in South Carolina have been identified as candidate sites for the Southeastern Campaign because of their large burn units (>500 ha), smoke management relevancy, increased burn opportunities, prescribed burning history, and ability to host a large operational burn in older fuel complexes that will contribute to large plume development (Figure 4b). Fort Stewart’s prescribed burn program rivals those of most other military installations and federal agency management units within the southeastern United States in annual area burned. The Savannah River site also regularly conducts large operational burns (i.e., to accomplish management objectives) in a completely secure facility with access to decades of high quality meteorological data. Vegetation and fuels are similar on both sites with plantation-established longleaf/slash pine stands that are generally burned every 1 to 4 years. FASMEE will target stand units with longer time since fire (4 to 6 years), resulting in fuel loads of greater than 30 Mg ha^−1^.

## 3. Coordinated Measurements

**Figure 5.**
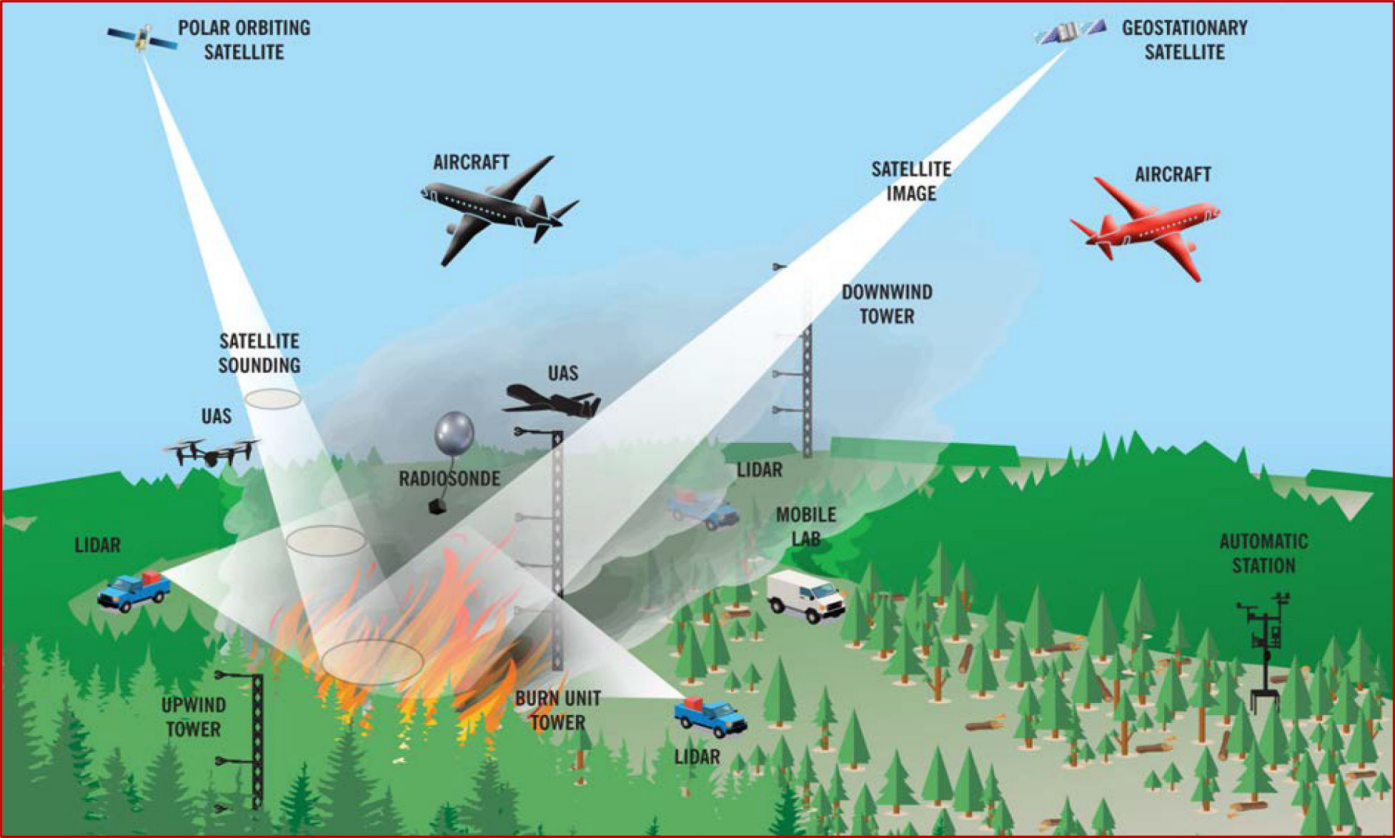
Schematic of coordinated observational campaign for integrated measurements of active fire behavior, plume dynamics, and smoke dispersion.

Each field campaign will contain coordinated and interrelated measurements of fuels and fuel consumption, fire behavior and energy release, fire meteorology and plume dynamics, and smoke production and chemistry (Figure 5). Producing high-quality datasets involves quality assurance, archiving, and documentation, as well as data cross-compatibility among the different measurements and platforms. In particular, measurements must be synchronized across time and space. This is especially critical for multi-temporal measurements of the fire and plume, for which failure will jeopardize the end-product usability. A key feature of the proposed field campaigns is that they will be designed up-front to be completely integrated with high-resolution mapping of fuels, fuel consumption, fire behavior, plume dynamics, and smoke measurements and temporally synched to provide context for related measurements (e.g., flaming fire front, heat release, and plume dynamics).

The following sections provide an overview of the sampling plan for each of the measurement disciplines: Fuels and Consumption, Fire Behavior and Energy Release, Plume Dynamics and Meteorology, and Smoke Chemistry and Transport. Any field campaign involving wildland fire must address a number of critical logistics, including safety and data quality. Ensuring safety throughout the project requires strict command structures, incident action plans, and aircraft safety plans, as well as site access and logistical staging plans. Detailed specifications and recommendations for operations, safety, and geospatial data management that synchronizes high-resolution spatial and temporal datasets are provided in the full study plan [20].

### 3.1. Fuels and Consumption

Fuels are the primary independent variable for all other FASMEE disciplines. Specifically, the rate of fuel consumption determines the heat release rate and other aspects of fire behavior, plume dynamics, and the gaseous and particulate composition of smoke emissions. Fuel consumption is most explicitly linked to fire behavior at fine scales of variability, but it also drives plume dynamics at coarser scales and spatiotemporal variation in smoke near the source [21]. Because smoke models require spatially and temporally resolved measures of fuel consumption to predict heat release rate and emissions, observational campaigns that involve fire–atmosphere interactions and smoke need to collect pre- and post-burn fuels information for all combustible material (trees, shrubs, forbs, grasses, coarse and fine woody debris, litter, and duff) [22]. Spatiotemporal characterization of preburn fuels and consumption is a key feature of FASMEE and offers a critical advance in approaches to source characterization and identifying sufficient resolutions for the modeling of smoke emissions from wildland fires.

For each site, we will couple ground-based measurements (e.g., loading, height, and day-of-burn fuel moistures) with remotely sensed 3D datasets to allow observations to be scaled from fine-scale inputs for physics-based, fire behavior models (WFR-SFIRE-CHEM, WFDS, FIRETEC) to coarser-scale data collection required by traditionally used fire and smoke models (e.g. FlamMap, BehavePlus, CONSUME, FOFEM, Daysmoke). Ground-based field sampling is the most reliable method for collecting the pre- and post-fire fuel measurements, but to capture heterogeneous fuel distributions, terrestrial and airborne LiDAR and structure-from-motion (SfM) methods for complementary point cloud data are also needed [22–24]. Key measurements include fuel load, architecture, composition (type), status (live/dead), and moisture. Fuels will be measured within a hierarchical sampling scheme using airborne laser scanning (ALS), terrestrial laser scanning (TLS), multi-spectral imagery, microwave imagery, photogrammetry, and non-destructive and destructive ground measurements based on inventories, subplots, and transects. Sampling across a range of spatial scales will provide an important sensitivity analysis of the scale of observations that are needed for the models of interest and how to quantitatively model 3D fuel properties from fine to coarser scales based on remotely sensed imagery [25–27]. Airborne LiDAR will be used to provide synoptic coverage of forest canopies and, with less sensitivity, the lower-level and surface vegetation layers [28]. At smaller scales, terrestrial LiDAR and multi- or hyperspectral imagery from unmanned aircraft systems (UAS) and/or towers/tethered balloons will be used for higher resolution fuels mapping [24–26]. Surface fuel components and fuel properties will be intensively sampled within Highly Instrumented Plots (HIPs) in each operational prescribed burn.

FASMEE also requires a high-resolution characterization of fuel consumption and heat release rates. The rate of fuel consumption per unit area relates more directly to combustion and heat release than fuel load, and consumption by combustion phase differs greatly by fuel component. Therefore, fuel consumption by type (e.g., live shrubs and grasses, fine wood, coarse wood, litter and organic soils) will be mapped, using a combination of active fire infrared (IR) imagery and ground-based post-fire sampling, to provide more direct relationships to energy flux and emissions than maps of fuel loads and vegetation type [27]. Characterizing the type of fuels can also identify the sources of flaming and smoldering consumption. For example, coarse wood and duff on site would be expected to contribute most to short- and long-term smoldering. Coupled active fire IR measurements with mapped fuels can be used to confirm sources of smoldering fuels. To provide evaluation datasets of existing operational models of consumption, we will compare gridded, mapped fuel consumption to unit-based predictions made by CONSUME [29] and FOFEM [30].

### 3.2. Fire Behavior and Energy

The Fire Behavior and Energy (FBE) discipline will address key questions related to the transfer of mass and energy between the fire and plume. Together with fuels and meteorology, fire behavior and energy determine the heat source for plume rise [31]. Spatially and temporally resolved fire behavior measurements also provide a critical context for post-fire ecological assessments including tree mortality and vegetation recovery [32]. Fire behavior and energy measurements will include quantitative fire radiation from satellite, airborne, and tower-based platforms. Other critical observations will include in-situ measurements (i.e., ground based, often in flame) describing flame-front dimensions, spread rates, radiative and convective heat fluxes, flame energy transport, emissions fluxes and combustion efficiency, and emissions partitioning between flaming and smoldering combustion. These measurements, in combination with those from the Fuels and Consumption discipline, will serve to support the use of high-resolution fire models such as WFDS and FIRETEC to provide plume models with accurate spatiotemporal maps of convective (i.e., sensible) heat fluxes from flame fronts.

Fire radiation mapping is foundational to understanding fire and plume development and to support plume modeling. Multiple measurements of fire radiation, using ground-based and airborne platforms, will be collected to derive spread rates and combined with in situ measurements, ground measurements, and modeling to derive flame-exit gas temperatures, velocities, and convective fluxes needed as inputs to plume models. The spatial and temporal characteristics of heat release is the most important determinant of plume dynamics and smoke transport [33]. Cross-scale measurements of fire radiation (i.e., from fuel cells to interacting flame fronts and plume structures) will help to answer questions about the genesis and evolution of spatial structure in plumes. Airborne measurements of fire radiation are generally required to sufficiently capture the high spatial and temporal resolution of fire behavior and energy release [34–36].

Coordinated measurements will be stratified across their expected range of variability within and among fires, and heat release rate will be characterized across spatiotemporal scales. When surface fires are measured from beneath a forest canopy, airborne heat release measurements must include estimates of fire radiation attenuation [28,37]. With canopy characteristics provided by the Fuels and Consumption discipline, established software can be used to model canopy radiation transmission and interception. Airborne radiation measurements, which allow the characterization of both fire energy and fire front location and spread, will provide critical information for improving satellite measurements of fire energy [35,36,38]. Currently, polar-orbiting satellites operating at 350 m to 1 km spatial resolution (e.g. VIIRS, MODIS) are used to estimate fire energy in mapping emissions [35]. Despite some specific drawbacks due to spatial resolution and limited temporal sampling (i.e., polar orbiters only pass over a site once or twice a day), these satellite-derived products, hold the promise of providing fire energy metrics valuable for other aspects of fire modeling, including informing models of fire behavior and plume dynamics [38]. While the rate of spread can be mapped from high quality, though qualitative, radiation measurements, cross-scale mapping of radiative power and energy and other derived products such as fire intensity and fuel consumption require quantitative measurements [32].

### 3.3. Plume Dynamics and Meteorology

Coupled fire–atmosphere models are capable of resolving the spatial distribution and temporal dynamics of fire behavior, fuel combustion, and smoke production. However, a more thorough understanding of plume dynamics and meteorology is needed, as well as evaluation datasets that can be used to assess existing models and inform the development of new operational models [39]. Fuel and fire dynamics determine how individual fires develop and merge into smoke plumes. How well these processes can be simulated by operational models and run under the constraint simulations that run faster than real time are major challenges for future operational model development. For example, multiple plume cores not only increase the horizontal heterogeneity of smoke but also change the entrainment of air into the plume, thereby modifying smoke plume rise and vertical distribution [33]. The formation and evolution of smoke updrafts are related to fuels and fire dynamics, including the ignition patterns and progression, fuel consumption, fire spread, and intensity.

Plume dynamics and meteorology measurements will be organized by four measurement platforms, including airborne, tower-mounted, ground-based in situ observations, and ground-based remote sensing. The atmospheric environment surrounding the burn unit and region is best characterized with a network of surface stations. Vertical atmospheric profilers will be placed around both the experimental burn unit and outside the experimental region to quantify local and mesoscale circulations and to provide measurements of atmospheric stability and wind shear. Key meteorological variables to characterize fire weather conditions and surface meteorology will include ambient air temperature, humidity, near-surface wind speed and direction of ambient and fire-induce wind fields [39]. Plume rise, entrainment, and fire-atmospheric circulations associated with the plume will be measured using in situ towers (e.g., [40]) and ground-based scanning Doppler LiDAR systems (ground and/or aircraft) [40–42], and a scanning Doppler dual-polarized, Ka-band radar system. Radars with dual-polarization capability have been shown to be useful for observing smoke plume structures and plume microphysics [43]. Monitoring the 3D structures of wildfire plumes requires the use of multiple LiDAR and radar systems to scan the entire plume simultaneously. UAS, radiosondes, and LiDAR are also needed to observe the plume dynamics and thermodynamics from above the canopy upward to several kilometers. To fully characterize plume structures and provide evaluation datasets for plume-rise and coupled fire–atmosphere models, measurements of 3D winds and temperature will be sampled within the plume from near the surface (or just above the fire front) to the top of the plume using towers and remote sensing instruments. Tall tower installations allow multiple temperature sensors and anemometers to be placed within and above the canopy. Above this level, remote sensors such as Doppler LiDAR can measure the winds within the plume. Additional airborne platforms such as UAS will sample the middle plume region to collect both kinematic and thermodynamic measurements.

### 3.4. Smoke Chemistry and Transport

Over the past decade, comprehensive laboratory and field experiments have significantly increased our knowledge of the composition [44,45] and processing of wildland fire emissions (e.g., [46–48]). With the exception of RxCADRE [16], previous field studies (e.g., SEAC4RS and BBOP) lacked the comprehensive fuels, fire behavior, and meteorological measurements that will be obtained by FASMEE. The type of pollutants and intensity of their emissions depends on the relative mix of flaming and smoldering combustion. Knowledge of the fuels consumed (fuel type, quality and quantity) and the relative importance of flaming and smoldering combustion are critical for developing emission factors that can be applied to different fuel and combustion conditions. This specificity in fuel types and combustion phases is also needed for the fuel consumption models used to manage prescribed burns and predict wildfire emissions such as CONSUME and FOFEM.

Full emissions characterization requires coordinated sampling of the plume and unlofted emissions from residual smoldering. Of the many emission studies of wildland fire conducted in the US, comprehensive ground measurements of emissions were lacking for all but a few of the units in the SCREAM study [49] and RxCADRE [50], which were both low intensity understory burns in southern US pine forests with low amounts of surface biomass. Currently, there are few observational datasets of emissions from forest fires in moderate to heavy surface or canopy fuels. Those that do exist have substantial deficiencies: limited chemical speciation of emissions, only cursory fuels information, and no measurements of un-lofted smoke. Concurrent measurements of the convective smoke plume and un-lofted smoke from residual smoldering combustion are needed to characterize the composition and emission intensities associated with fires that occur in wildland fire events in fuels with moderate to high fuel loadings.

The magnitude of pollutant emissions from wildland fires varies greatly depending on fire size (area burned), fire duration, fuel characteristics, combustion efficiency, and meteorological conditions [44,51]. Chemical production from fires is complex, highly variable, and often difficult to predict [52]. Typically, 3D photochemical grid models such as the Community Multiscale Air Quality Model (CMAQ) or Comprehensive Air Quality Model with extensions (CAMx) are used to estimate local to continental scale ozone (O_3_), particulate matter (PM), and regional haze for scientific and regulatory assessments. Field and laboratory data from specific and well characterized wildland fires are important to improve emissions estimation approaches, and for assessing plume transport and chemical evolution in photochemical transport models [53–57]. This will enhance confidence in fire predictive capabilities to support future scientific and regulatory assessments related to fire impacts on local to continental scale O_3_, PM, haze, climate, and air toxics.

A comprehensive characterization of smoke emissions in FASMEE will require multiple instruments and techniques employed from both ground-based and airborne measurement platforms. To understand chemical evolution in the smoke plume, detailed precursor and chemical product measurements are needed in the near field to define emission rates of key precursors as well as at different downwind distances (ideally even hundreds of kilometers from the fire source) extending over a multi-day period. Smoke and emissions measurements will be collected in three tasks, organized by vertical sampling range: Lofted plume sampled via manned aircraft, intermediate-level smoke sampled from UAS or tethered balloon (aerostat), and sub-canopy smoke collected with a ground-based instrumentation package and towers. Airborne chemical measurements from a manned aircraft are required to sample emissions in the lofted plume and to characterize the chemical processing of the emissions as the plume mixes with the ambient atmosphere and is transported downwind of the source. Flight profiles will provide intensive horizontal and vertical sampling of the near-field smoke plume (30 km from source).

The primary purpose of the intermediate-level smoke measurements is to monitor fresh emissions while the manned aircraft is sampling downwind. Many of the factors that influence the combustion process (the mix of flaming and smoldering emissions), and the overall chemical composition of fresh smoke, may vary considerably over the lifetime of a fire. Although the manned aircraft will conduct pseudo-Lagrangian sampling (e.g., [48,58,59]), a continuous near-source measurement of fresh emissions will be critical for interpreting the downwind observations when the source characteristics are changing rapidly. The intermediate-level smoke sampling will provide continuous measurements of modified combustion efficiency and short-duration batch samples of PM and key volatile organic compounds. The secondary purpose of the intermediate-level smoke sampling is to measure the vertical distribution of smoke between the canopy and the lowest level of manned aircraft measurements. Aerostat and UAS are both suitable for the intermediate-level smoke sampling, but FASMEE will favor UAS use due to its greater mobility and ability to sample significantly larger volumes of smoke than aerostats.

Sub-canopy sampling will use ground-based platforms to characterize emissions over the life cycle of the burns. Direct emissions from biomass burning are a complex mixture of gases and aerosols. The emission factors measured must represent all phases of combustion, including flaming, near-term smoldering, and long-term smoldering phases. Four types of ground-based platforms will be used to measure buoyant smoke being entrained into the convective plume, unlofted smoke (drift smoke), and post-fire-front extended smoldering. The subcanopy plan includes instrumented towers erected within the burn unit, which will sample smoke forming and entraining into the bottom of buoyant plume and drift smoke. Unlofted drift smoke will be measured using fixed-site subcanopy instrument packages (SIP) located immediately downwind of the unit, flanking a mobile lab (ML). If available, a SIP will be located upwind of the burn unit to monitor background conditions. The post-fire-front sampling will use one or two mobile instrument packages (MIP; defined as a package that could be carried by two people within the burn unit). The post-fire-front sampling will measure emissions from independently smoldering fuel components (e.g., logs, stumps, and duff mounds). The SIP will also operate for an extended duration and sample smoke from extended smoldering that drifts across the sites.

## 4. Application to Model Evaluation and Development

As wildland fire, smoke dispersion, atmospheric chemistry, and global climate models have become more sophisticated, there is an increasing need for complex datasets that are coordinated and comprehensive, from ground-based observations of fuels and fire behavior, to near-source plume dynamics and emissions, smoke trajectories, dispersion, and atmospheric chemistry. FASMEE is designed to provide these integrated observations and to serve as a template for other future campaigns that together will provide the evaluation datasets necessary to evaluate and develop next-generation, operational fire and smoke modeling systems. Many models that drive today’s operational systems need comprehensive validation and evaluation, as well as an understanding of a model’s predictive capability over the full range of wildland fire environments and weather conditions [60]. Improving estimates of plume rise and smoke production and dispersion is fundamentally based on characterizing fire–atmosphere interactions, including wildland fire behavior and plume dynamics [61,62]. Estimates of smoke emissions and dispersion are highly dependent on accurate characterization of many interrelated variables including area burned, pre-burn biomass, fuel structure and composition, fuel consumption by combustion phase, fire behavior, heat release, plume dynamics, meteorology, and smoke chemistry [63]. More details on fire and smoke models needs and the FASMEE observations planned to provide coordinated datasets for model evaluation and development are provided in a companion article [31].

Operational applications require that the models be executed rapidly enough to provide usable predictions and that the data needed for their executions are readily available. Existing models that predict area burned, fuel consumption (CONSUME and FOFEM)[29,30], fire behavior (Behave, FlamMap)[64–67], smoke transport and dispersion models (Daysmoke, VSmoke, HYSPLIT) [8,68,69] span a broad range of complexity. In general, these systems have been simplified to be used in wildland fire management applications (e.g., fuel consumption, wildland fire spread, and smoke prediction for tactical and strategic planning purposes) and rely on sets of assumptions and algorithms derived from observations and theory. Specifically, operational fire and smoke modeling systems rely on simplified fuel consumption, fire behavior, heat release, and plume models that are based on empirical or statistical relationships developed from laboratory, field, and smokestack observations [70,71]. Contemporary fire and smoke operational systems generally run ensembles of calculations from simplified models of fire behavior, consumption to smoke production, and dispersion (e.g., FlamMap, Behave Plus, CONSUME, FOFEM, FEPS, V-smoke, Daysmoke, PB Piedmont, BlueSky, WFDSS, IFTDSS, Table 3). Ensembled model predictions have associated errors and uncertainty along each modeling step that are generally not characterized or quantified.

**Figure 6:**
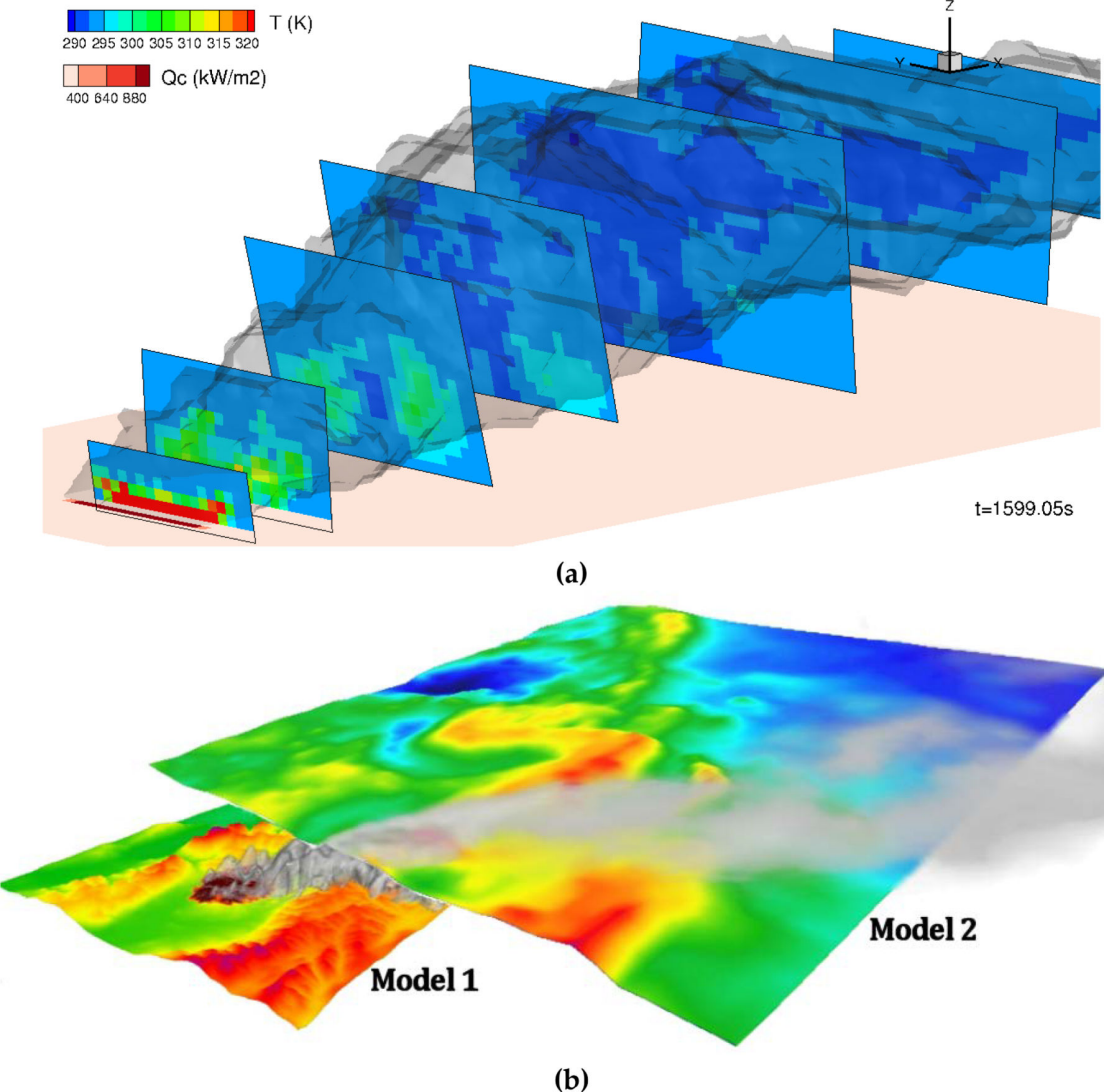
**(a).** Example of smoke plume modeled using the Wildland-urban-interface Fire Dynamics Simulator – full physics based model (WFDS-PB), a coupled fire–atmosphere model that uses a simplistic representation of fuels and fire behavior to estimate fire progression and energy release. A line source is used and cross-sectional planes are shown; the planes are orthogonal to the centerline plume motion at each point downwind. **(b)**. Example of how a physics-based fire and plume model (Model 1) can drive a coarser-scale air quality/smoke chemistry model (Model 2). Model 1 has a small (kilometers), high-resolution (tens of meters) grid that acts as input to the larger (hundreds of kilometers), coarser (kilometer) grid. In this way, the plume is explicitly captured and “handed off” to the broader air quality model.

Due to the computational limitations of physics-based fire–atmosphere models, it remains challenging to implement them in operational systems useful to wildland fire managers. However, future operational systems may offer the ability to develop a new generation of models that are calibrated with fully dynamic models and provide superior accuracy across a wider range of conditions thanks to a combination of simplified physics and parameterizations. As the resolution of the weather forecasting models increases, these fire–atmosphere interactions and smoke plumes become explicitly resolvable on fine-model grids, which opens new avenues for development of new operational models explicitly resolving plume dynamics that inform operational models at coarser resolutions (Figure 6).

### 4.1. Past Fire–Atmosphere Field Campaigns—Lessons Learned and Legacy Datasets

Since the 1990s, a range of field-based large fire experiments have been conducted across major biomes and regions of the globe to study wildland fire behavior, fire–atmosphere interactions, including plume dynamics, and smoke dispersion. To compile lessons learned and legacy datasets, the FASMEE planning team synthesized these studies and source datasets of past studies that FASMEE observations will augment (see Supplementary Materials).

One of the earliest integrated fire–atmosphere observational campaigns was the International Crown Fire Modeling Experiment (ICFME), initiated to evaluate fire propagation and spread in boreal landscapes and the role of boreal wildfires in ecosystem function and global climate change [78,79]. The ICFME field campaign began in 1993 with the Bor Forest Island Fire Experiment, a 49-ha fire in an island of mature Scots pine (*Pinus sylvestris*) in north-central Siberia [80]. This experiment studied fire behavior in a high-intensity, stand-replacement fire in addition to fire meteorology, trace gas and aerosol emissions, and long-term fire effects and recovery. From 1997 to 2000, 18 burns were conducted at an ICFME site in the Fort Providence area of the Northwest Territories of Canada [79]. Observations were collected by over 100 collaborators and included measurements of radiant emissive power, wind and temperature fields, crown fire behavior, black carbon, fuel consumption, trace gas and aerosol formation, and firefighting and structure protection strategies in boreal wildfires. In July 1999, the project FROSTFIRE conducted an 809-ha experimental burn in mixed spruce and hardwood boreal forests near Fairbanks, Alaska [81]. Over 50 research teams conducted integrated sampling and measured fire behavior, fire effects, energy fluxes, and trace gas emissions. Datasets from the ICFME are still used today and highlight the importance of these integrated experiments and the legacies of the studies for model development and validation.

In a project on sub-canopy transport and dispersion of smoke, five experimental units were burned as low-intensity surface fires in mature longleaf pine stands in the southeastern United States (Calloway Forest/Sandhills Preserve, North Carolina). This study evaluated sub-canopy emissions and smoke dispersion from low-intensity prescribed burns that are common in the southeastern US, and the results collectively contribute to better understanding of regional air quality issues [50]. Observations included pre-fire fuels, consumption and emissions, plume rise, and dispersion and were used to evaluate predicted smoke dispersion in the BlueSky Framework [63].

The FireFlux I and II experiments collected field observations of fire–atmosphere interactions to evaluate coupled fire–atmosphere models including WRF-SFIRE [82] and MesoNH/ ForeFire [5]. Observations included micrometeorology of the fire-front passage and plume thermodynamics simultaneously collected during wind-driven grassland fires using the same burn location [83–85].

The Prescribed Fire Combustion and Atmospheric Dynamics Research Experiment (RxCADRE) field campaign was conducted in the southeastern United States [16]. The project was inspired by a series of meetings held by the Core Fire Science Caucus. This was a group of 30 fire scientists who identified critical fire research needs and agreed to support integrated, multi-scale observations of fire and smoke. From 2008 to 2012, RxCADRE conducted several experimental prescribed burns to collect coordinated, multi-scaled observations of fuels, meteorology, fire behavior, radiative power and energy, emissions and fire effects. Burn sites in grassland, grass-shrub, and managed forests of the southeastern United States were selected because of the frequent use of prescribed burning in the region, which not only made the project areas highly relevant but also ensured that prescribed burns would be frequent enough to support the experiments. Interdisciplinary scientists collected observations before, during, and after each burn, with the goal of obtaining co-located measurements that could support multiple fire-related disciplines. Measurements and preliminary results were published in an International Journal of Wildland Fire special issue, and data were made available on a globally accessible Research Data Archive maintained by the United States Department of Agriculture, Forest Service [86].

Building on the success and lessons learned from RxCADRE, the FASMEE campaign was launched to provide integrated measurements of higher-intensity fire behavior and smoke. FASMEE takes advantage of lessons learned from past campaigns including the importance of involving fire scientists and model developers at the inception of measurement campaigns and the critical value of spatiotemporal synchronization of all measurements. Full funding for the FASMEE campaign has yet to be secured, but the study plan and conceptual promise of integrated measurements provides a compelling case for investment in large-scale, comprehensive measurement campaigns of fuels, fire and smoke production.

The FASMEE team is closely collaborating with the 2018 Western Wildfire Experiment for Cloud Chemistry, Aerosol Absorption and Nitrogen experiment (WE-CAN) and the joint NASA and NOAA FIREX-AQ campaign planned for summer 2019 to provide source characterization for the planned airborne sampling campaigns to study emissions, chemical evolution, transport, and impacts of wildland fires. If possible, the timing of FASMEE prescribed burns will be coordinated with FIREX-AQ sampling to provide a detailed source characterization and greatly expand the information gained on emissions and smoke transport. The FASMEE burns will also offer unique opportunities for post-fire ecology research including tree mortality and vegetation response studies that make use of mapped fuels consumption and characterization of fine-scale fire behavior. Advances in remote sensing interpretation can also be made by pairing remotely sensed imagery such as Synthetic Aperture Radar and thermal infrared to assess fuel moisture, consumption, fire spread rates and energy release and hot-spot assessment.

## 5. Summary

The past few decades have brought enormous advances in not only our understanding of fire and smoke dynamics but also our ability to observe and model fuel consumption, fire behavior, plume development, smoke production, and dispersion. Operationally, the adoption of fire and smoke models has been rapid. Advanced model output is now routinely available to managers; examples include the incorporation of fire models and the adoption of BlueSky-based smoke impact model runs within the Wildland Fire Decision Support System (WFDSS) and the Interagency Fuel Treatment Decision Support System (IFTDSS, currently under development).

Although these systems provide value-added information, they are also relatively simplistic in their treatment of complex fire dynamics and therefore have issues in how well they can perform. More complex models, such as the coupled fire–atmosphere–chemistry models (e.g., WRF-SFIRE-CHEM, WFDS and FIRETEC), are in testing to assess their ability for operational use. The advances to date offer the promise of capturing the underlying dynamics of fire–atmosphere interactions, but they also require greater input data. Unfortunately, a lack of observational data means substantial uncertainty about what the underlying dynamics are, and how well such systems can represent them. FASMEE is designed to facilitate the transition of more advanced modeling systems into operational use by supplying critical data necessary to facilitate improvements, testing, and adoption.

The expected outcomes from the FASMEE project include (1) improved scientific knowledge of the physically coupled fuels–fire–smoke–chemistry system; (2) exportable methodologies for measuring fuels for fire spread, fuel consumption, and fire emissions models; (3) new insights concerning the processes that drive the spatial organization of fire energy and emissions that defines the transition between fires and plumes that impact air quality; and (4) improvement of existing operational fire and smoke models and the development of new, more advanced models based on the collection of an unprecedented dataset (fuels, fire, meteorological, smoke plume and chemistry).

The FASMEE campaigns are specifically planned to provide a full referenced set of synchronized fuels characteristics and fire behavior measurements for future campaigns and other related disciplines. For example, the proposed measurement campaigns offer a unique opportunity for related post-fire ecology studies that take advantage of highly resolved pre-burn vegetation and combustion mapping and associated datasets. Documentation of FASMEE burns will include spatiotemporal mapping of fuels, fuel consumption, fire behavior, and plume development to facilitate broad use of the datasets collected in the completed campaigns. The FASMEE data archive is being designed by a geospatial data manager and coordinated with partner agencies to ensure the development of a legacy dataset that can be amplified in subsequent coordinated field campaigns.

## Supplementary Material

Supporting Information

## Figures and Tables

**Table 1.** Coupled fire–atmosphere models and atmospheric models that can be evaluated with Fire and Smoke Model Evaluation Experiment datasets. Platforms and decision support systems or other applications that house specific models are presented in the “Applications” column.

Model	Description	Applications	FASMEE Datasets	References

CAWFE	Coupled Atmosphere-Wildland Fire-Environment (CAWFE): a coupled weather—wildland fire computational model.	NCAR Simulation model (Janice Coen)	Fire behavior, meteorology and plume dynamics.	[7]
FIRETEC	HIGRAD/FIRETEC: physics-based, 3-D model that represents the coupled interaction between fire, fuels, atmosphere, and topography.	Simulation Model, Los Alamos National Laboratory, included in STANDFIRE	Fuel consumption, gridded fire behavior and radiative energy, meteorology and plume dynamics.	[9]
MesoNH/ForeFire	Mesoscale non-hydrostatic model coupled with a surface atmospheric interaction model (SURFEX).	Desktop (unix)	Meteorology and plume dynamics	[5]
Vesta	Large-scale, cell-based wildland fire simulator developed within the Fire Paradox project.	Desktop	Gridded fire behavior and fire radiative energy observations.	[11]
WFDS	Wildland-Urban-Interface Fire Dynamics Simulator: computational fluid dynamics model that resolves buoyant flow, heat transfer, combustion, and thermal fuel degradation.	Desktop (unix) STANDFIRE (under development)	Fuel consumption, gridded fire behavior and radiative energy, meteorology and plume dynamics.	[10]
WRF-SFIRE (Spread FIRE model)	Weather Research and Forecasting—Spread Fire: combined atmosphere and fire spread model.	High performance computing cluster	Gridded fire behavior, meteorology and plume dynamics.	[3,4]

**Table 2.** Active and planned FASMEE Campaigns.

Campaign and Timeline	Potential Sites	Description

Coordination with WE-CAN and FIREX-AQ large aircraft campaigns (July–August 2018 and 2019)	US wildfires and prescribed burns	FASMEE is collaborating with two large-scale campaigns to provide source characterization for emission studies from western wildfires (WE-CAN, https://www.eol.ucar.edu/field_projects/we-can) and US wildfires and prescribed burns (FIREX-AQ)
Southeast (planned)	Fort Stewart Savannah River Site	Highly instrumented prescribed underburns completed in managed pine forests with heavy surface fuel loads, ignited for a moderate-intensity fire
Southwest (planned)	Fishlake National Forest Kaibab National Forest	Moderately and highly instrumented prescribed burns in dense mixed conifer-aspen forests, ignited for a high-intensity, stand-replacement fire

**Table 3.** Operational fire models currently in use for wildland fire management in the United States that can be evaluated with FASMEE datasets. FFE-FVS = Fire and Fuels Extension of the Forest Vegetation Simulator, IFTDSS = Interagency Fuel Treatment Decision Support System, WFDSS = Wildland Fire Decision Support System.

Model	Description	Applications	FASMEE Datasets	Reference

BehavePlus	Models surface and crown fire spread and intensity, safety zone and point source size, fire containment, spotting distance, crown scorch height, tree mortality, and probability of ignition.	Desktop FFE-FVS Fire Family Plus Wildland Fire Decision Support System	Fire intensity; spread rate	[64,65]
CONSUME	Predicts consumption and emissions by combustion phase and fuelbed category.	BlueSky Fuel and Fire Tools IFTDSS	Consumption by category: flaming, smoldering and long-term smoldering combustion.	[29]
DaySmoke	Models smoke transport and dispersion.	Desktop	Plume rise; short-term smoke transport	[8]
FARSITE	Fire Area Simulator (FARSITE) spatially and temporally simulates fire spread and behavior under heterogeneous conditions.	Desktop WFDSS	Fire area & perimeter; spread rate;	[72]
FireFamily Plus (FFP)	Fire climatology and occurrence program; summarizes and analyzes weather observations and computes fire danger indices.	Desktop	Meteorological observations	[73]
First Order Fire Effects Model (FOFEM)	Predicts tree mortality, fuel consumption, smoke production, and soil heating.	Desktop IFTDSS module	Consumption by category; tree mortality; soil heating.	[30]
Fire Simulation Model (FireSim) Fire Spread Probability (FSPro)	Geospatial probabilistic model that predicts fire growth; designed to support long-term decision-making.	Desktop WFDSS module	Fire area; perimeter	[74]
FlamMap	Fire behavior mapping and analysis program that computes potential fire behavior characteristics.	Desktop IFTDSS, WFDSS	Fireline intensity; spread rate.	[67]
WindNinja	Computes spatially varying wind fields for wildland fire application.	WFDSS module	Gridded wind fields	[75]
HYSPLIT	Computes simple air parcel trajectories, as well as complex transport, dispersion, chemical transformation, and deposition simulations.	BlueSky; desktop; atmospheric modeling systems.	Smoke dispersion	[69]
CALPUFF	Non-steady-state meteorological and air quality modeling system.	Desktop	Meteorology; plume rise; smoke dispersion	[76]
CMAQ	Eulerian chemical transport model treating all emission sources, transport, chemical transformation, and deposition processes to estimate 0_3_, speciated PM_2.5_, and toxics.	Unix-based computer system	Meteorology; plume rise; smoke dispersion; smoke chemistry	[77]
VSmoke	Smoke dispersion model to estimate prescribed fire impacts	Web-based Desktop	Plume rise; smoke dispersion	[68]
