# Supplementary material for: The Fire and Smoke Model Evaluation Experiment—A Plan for Integrated, Large Fire–Atmosphere Field Campaigns
Source: Atmosphere (Basel). Author manuscript; Available in PMC 2020 Jul 23. (PMC7376818; doi:10.3390/atmos10020066)
Supplement: Supporting Information [file NIHMS1600245-supplement-Supporting_Information.docx]

**Supplementary Appendices**

Supplementary tables include summaries of major field data collection campaigns of wildland fire and smoke. Table S1 summarizes field-based experiments from the 1970s to 2000. Table S2 summarizes major airborne sampling campaigns as part of the Biomass Burning Experiment (BIBEX) projects lead by the International Geosphere-Biosphere Program. Table S3 includes recent field campaigns that collected observations of fire-atmosphere interactions (FAI).

**Table S1**: Major field-based experiments on fire behavior and smoke from the early 1970s to 2000, study dates, general measurements and study location, and application to fire and smoke modeling. BIBEX burns are summarized in Table S2.

| **Project and key references** | **Study dates** | **Measurements and location** | **Model application** | **Source references** |
| --- | --- | --- | --- | --- |
| Black spruce crown fire studies | 1973 to 1983 | Experimental fires were conducted in jack pine (immature and mature) forests in north central Ontario. | Dataset used to evaluate the Canadian Forest Fire Weather Index System. | Albini and Stocks (1986)  Stocks (1980)  Stocks (1987a)  Stocks (1987b)  Stocks (1989) |
| International Crown Fire Modeling Experiment (ICFME) | 1990 to 2000 | Circumboreal forests (see below) | Validation of empirical crown fire spread and intensity model. | Alexander *et al.* (1998)  Butler *et al.* (2004)  deGroot *et al.* (2004)  Putnam and Butler (2004)  Stocks *et al.* (2004b) |
| - ICFME - Bor Forest Island Fire Experiment. Fire Research Campaign Asia-North (FIRESCAN) | July 1993 | Pre-fire vegetation, fuel, fire behavior, atmospheric emissions and post-fire effects in scotch pine, western Siberia |  | FIRESCAN Science Team (1996) |
| - ICFME (Canada) | 1997-2000 | Crown fire behavior, aerosol and trace gas formation, and post-fire regeneration using 18 experimental burns in the Northern Territories, Canada | Contributed to development of crown fire behavior models. Used to research the inclusion of wind data in FIRETEC. | Stocks *et al.* (2004a) |
| - ICFME FROSTFIRE (Alaska) | July 1999 | Wind, temperature fields, crown fire behavior, fuel consumption, radiant energy, and trace gas and aerosol formation in a high-intensity burn of boreal spruce-hardwood forests, Fairbanks, Alaska | Assisted development of fuel characteristic classes (FCC), which drive CONSUME and EPM. | Harden *et al.* 2004 |
| Australian Fire Experiments | 1990-2000 | Various studies to evaluate fire behavior and fire and vegetation dynamics. | Contributed to development of wildfire spread and tree population dynamics models. |  |
| - Annaburroo | 1985-2001 | Fire behavior and factors influencing fire spread in northern Australian grasslands. | Used in development of quasi-empirical wildfire spread rate model. | Cheney *et al.* (1993, 1998) |
| - Kapalga fire experiment | 1990-1994 | Fire behavior, atmospheric chemistry, hydrology, and Fire effects to vegetation and wildlife in eucalyptus savannas of northern Australia | Assisted development of Flames Simulation Model. | Williams *et al.* (1998)  Andersen *et al.* (1998)  Andersen *et al.* (2006) |
| - Project Vesta | 1998-2001 | Pre-fire fuel characterization, wind fields and fire behavior using 100 (4-ha burns in eucalypt forests of southwestern Australia. | Creation of Dry Eucalypt Forest Fire Model (aka Project Vesta Model). | Gould *et al.* (2008) |
| Crude Oil Burning Experiments | 1993-1994 | Pool fire experiments and plume characterization and dispersion. | Evaluation of A Large Outdoor Fire Plume Trajectory Model (ALOFT). | McGratton *et al.* (1997) |
| - Newfoundland Offshore Burn Experiment (NOBE) | Aug 1993 | Offshore burning experiment by Environment Canada. | Assisted development of the Large Eddy Simulation (LES) plume trajectory model. | Fingas (1995)  Ross *et al.* (1996) |
| - Burning of Emulsions Tests | Sept 1994 | Three emulsion burns conducted by Alaska Clean Seas (ACS) near Prudhoe Bay, Alaska. Measurements included plume development, atmospheric turbulence and smoke dispersion. | Used to evaluate the Large Eddy Simulation (LES) plume trajectory model. | McGratton *et al.* (1995) |
| - Open-pit diesel burning tests in Mobile Alabama (flat terrain) | Oct 1994 | Three mesoscale burns in open pans of known volume were conducted in Mobile Bay, Alabama. Smoke plumes, turbulence and particulate concentrations were measured. | Used to evaluate the A Large Outdoor Fire plume Trajectory (ALOFT) model. | Walton *et al.* (1995) |

**Table S2**: Biomass Burning Experiment (BIBEX) projects lead by the International Geosphere-Biosphere Program.

| **Project** | **Study dates** | **Measurements and location** | **Data and key references** |
| --- | --- | --- | --- |
| Southern Tropical Atlantic Regional Experiment (STARE) | 1990 to present | Sources and atmospheric transport of trace gases including O_3_ and CO in the southern tropical Atlantic Ocean | Andreae *et al.* (1996) |
| Transport and chemistry near the Equator-Atlantic (TRACE-A) | August-October 1992 | Western portion of STARE program including Brazil, Congo Republic and Ascension Island as well as aircraft observations spanning the South Atlantic. Trace gases including O3, CH_4_ and N_2_O. | Goldammer *et al.* (1997) |
| Southern Africa Fire-Atmosphere Research Initiative (SAFARI-92) | 1992 | Ground and airborne chemical and meteorological measurements in South Africa savannas. | Goldammer *et al.* (1997) |
| AFARI-97 Field Campaign in Kenya | Sept-Oct 1997 | Continuation of SAFARI in east African savannas in Kenya with an emphasis on CO, CO_2_ and aerosol formation. | Delmas *et al.* (1999) |
| SAFARI 2000 | 1999-2000 | Coordinated ground and airborne campaign to quantify emissions from dry and wet-season biomass burning. | Keene *et al.* (2006) |
| Zambian International Biomass Burning Emissions Experiment (ZIBBEE) | Jun-Aug 1996 and  Aug-Sept 1997 | Study of seasonal trends in biomass burning, emissions and aerosol formation in Zambia. | Hoffa *et al.* (1999) |
| Experiment for Regional Sources and Sinks of Oxidants (EXPRESSO) | Nov-Dec 1996 | Coordinated atmospheric measurements from research aircraft and NOAA-AVHRR imagery of dry-season burning in the Republic of Congo. | Delmas *et al.* (1999) |
| European Studies on Trace Gases and Atmospheric Chemistry (EUSTACH) | 1999 wet-to-dry and dry-to-wet season transitions | Integrated study of biogeochemical cycling of carbon, aerosols, trace gases, water and energy in the Amazon Basin of Brazil. | Andreae *et al.* (2002) |
| Cooperative LBA Airborne Regional Experiment (LBA-CLAIR) | Mar-April 1998  July 2001 | Trace gas emissions and aerosol formation from biomass burning in the central Amazon basin | Andreae *et al.* (2001) |
| Smoke, Aerosols, Clouds, Rainfall and Climate (SMOCC) | Dry season 2002 | Aerosol and cloud formation during dry season burning in the Amazon basin, Brazil. | Andreae *et al.* (2008) |

**Table S3**: Recent field campaigns that collected observations of fire-atmosphere interactions (FAI). For a review of FAI research see Potter 2012a, b).

| **Project** | **Study dates** | **Measurements and location** | **Model support** | **Data and key references** |
| --- | --- | --- | --- | --- |
| FireFlux I and II | Jan 2013 | Fine-scale turbulence and plume thermodynamics during an experimental burn of a 62-ha grassland unit in Houston, Texas. | Assessment of FOREFIRE/MESONH and WRF-Sfire. Validation of free-agent cellular automata model of fire spread and fire-atmosphere coupling. | Clements *et al.* (2007, 2008, 2010) |
| Sub-canopy transport and dispersion of smoke (Subcanopy) | Mar 2010-Feb 2011 | Pre-fire fuels characterization, fuel consumption, emissions and dispersion studied within five experimental burns in mature longleaf forests of North Carolina. | Evaluation of simple puff dispersion model for in-canopy plume transport and dispersion. Developed and validated pathway in BlueSky Smoke Modeling Framework for low-intensity/smoldering emissions and subsequent smoke forecasts. | Strand et al (2016) |
| Integrated Monitoring and Modeling System for Wildland Fires (IS4FIRES) | June 2009 | Ground-based and aircraft measures of emissions, aerosol formation and meteorological conditions within and downwind of the plume were collected in an experimental burn of a Scots pine logging slash site in Finland. | Validation of meso- to global-scale atmospheric models. System for Integrated Modeling of Atmospheric Composition (SILAM), Dispersion from strongly buoyant sources - Finnish Meteorological Institute (BUO-FMI), High Resolution Limited Area Model HIRLAM), and European Center for Medium-Range Weather Forecasts Numerical Weather prediction models. | Clements *et al.* (2009) |
| Development of modeling tools for predicting smoke dispersion from low-intensity fires (JFSP 09-1-04-1) | 2009 | Located in New Jersey Pine Barrens Administrative Area and National Reserve. Focus on models of smoke movement. | Test and evaluation of Weather Research and Forecasting Model Flexible Particle Dispersion Model (WRF-FLEXPART), Atmosphere to CFD (A2C), Regional Atmospheric Modeling System based Forest Large Eddy Simulation Model (RAFELS), and Advanced Regional Prediction System (ARPS). | Heilman *et al.* 2013 |

Table S4: Topic areas and measurements collected in the RxCADRE experimental burns in 2008, 2011 and 2012 at Eglin Air Force Base, Florida and Joseph W. Jones Ecological Center, Georgia. Data are available at: http://www.fs.usda.gov/rds/archive/Product/RDS-2015-0036

| Topic | Measurement | Reference |
| --- | --- | --- |
| Pre- and post-fire fuel characterization | Pre- and post-fire surface fuel loading and cover  Fuel height (terrestrial lidar)  Fuel consumption | Ottmar et al (2016)  Rowell *et al.* (2016)  Hudak *et al.* (2016) |
| Fire meteorology | Ambient fire weather conditions  Fire-atmosphere interactions  including fire-induced circulations, sensible heat flux and turbulence | Clements *et al.* (2016) |
| Fire behavior | Flame height and spread rate  Radiant and total energy  Convective heating and cooling | Butler *et al.* (2016)  O’Brien *et al.* (2016) |
| Fire radiative power and energy | Fire radiative power  Fire radiative energy and fuel consumption | Dickinson *et al.* (2016)  Hudak *et al.* (2016) |
| Smoke and emissions | Aerial and ground-based measures of CO_2_, CO, CH_4_, PM and black carbon  Optical properties of smoke | Strand *et al.* (2016) |

Table S5: Recent atmospheric chemistry measurement campaigns.

| Project | Study dates | Location and measurements | Model support | Data and key references |
| --- | --- | --- | --- | --- |
| Studies of Emissions and Atmospheric Composition, Clouds and Climate Coupling by Regional Surveys (SEAC4RS) | Ongoing | Near surface and tropospheric measurements of pollutant emission including gas and aerosol evolution and influence on meteorology and climate in the western United States. | Creation of cirrus database for evaluation and improvement of global climate models. Evaluation of AERONET retrievals used as input for climate and chemical transport models. |  |
| Arctic Research of the Composition of the Troposphere from Aircraft and Satellites (ARCTAS) | July 2008 | Opportunistic study of air-born smoke plumes in the arctic atmosphere. Two research aircraft collected emissions data from known plumes including source and downwind to evaluate the long-range transport of wildland fire smoke and compare to satellite observations. Based in Canada. | Validation of WRF-Chem model. Improved snow BRDF model. | Jacob *et al.* (2010) |
| Biomass-burning Aerosols in South East Asia: Smoke Impacts Assessment (BASE-ASIA) | Feb-May (dry season) 2006 | Regional study of atmospheric trace gases and aerosol formation and dispersion from biomass burning in central Thailand using coordinated satellite and ground-based measurements of emissions and transport. | Validation of the Community Multiscale Air Quality Modeling System (CMAQ) for regional chemical transport. | Tsay *et al.* (2013) |
| 7-SEAS/Dongsha Experiment | Mar-June 2010 | Collected observations of atmospheric chemistry, radiation and meteorology and aerosol properties from biomass burning on Dongsha Island, Taiwan. | Validation of HYSPLIT and NAAPS. | Wang *et al.* (2011, 2013) |
| South American Biomass Burning Analysis (SAMBBA) | Sept 2012 (dry to wet season transition) | Located in Porto Velho. Used a large research aircraft to make radiation, trace gas and aerosol measurements from biomass burning coordinated with satellite measurements. | Suggests over- and underestimation from models in eastern Amazon forest, including underestimation of smoke particle loading by 3BEM- and FINN-driven model simulations, overestimation of fire emission from 3BEM-FRP, and underestimation of atmospheric aerosol load by MACC and Coupled Aerosol and Tracer Transport Model. | Pereira *et al.* (2016)  <http://www.atmos-chem-phys.net/16/6961/2016/acp-16-6961-2016.pdf> |
| FIREX-AQ | Ongoing | Combining aircraft and in-situ measurements to study the chemistry and evolution of emissions from biomass burning using both nighttime and daytime sampling of North American wildfire plumes. | Will contribute to wildfire impact modeling including the effects of fire emissions on global chemistry and climate. Will also validate meteorological and chemical models. |  |

**References**

**References**

Albini, F.A.; Stocks, B.J. Predicted and observed rates of spread of crown fires in immature jack pine. *Combust. Sci. Technol.* **1986**, 48, 65-76.

Alexander, M.E.; Stocks, B.J.; Wotton, B.M.; Lanoville, R.A. An example of multi-faceted wildland fire research: the International Crown Fire Modeling Experiment. In *Proceedings of the 3rd International Conference on Forest Fire Research, Coimbra, Portugal, 16 November – 19 November 1998*.

Andersen, A.N.; Braithwaite, R.W.; Cook, G.D.; Corbett, L.K.; Williams, R.J., Douglas, M.M.; Gill, A.M.; Setterfield, S.A.; Muller, W.J. Fire research for conservation management in tropical savannas: introducing the Kapalga fire experiment. *Austral Ecol.* **1998**, 23, 95-110.

Andersen, A.N.; Hertog, T.; Woinarski, J.C. Long‐term fire exclusion and ant community structure in an Australian tropical savanna: congruence with vegetation succession. *J. Biogeogr.* **2006**, 33, 823-832.

Andreae, M.O.; Fishman, J.; Lindesay, J. The Southern Tropical Atlantic Region Experiment (STARE): Transport and Atmospheric Chemistry near the Equator-Atlantic (TRACE A) and Southern African Fire-Atmosphere Research Initiative (SAFARI): An introduction. *J. Geophys. Res.* **1996**, 101(D19) 23, 519– 23, 520.

Andreae, M.O., Artaxo, P., Fischer, H. *et al.* Transport of biomass burning smoke to the upper troposphere by deep convection in the equatorial region. *Geophys. Res. Lett.* **2001**, 28, 951-954.

Andreae, M.O., Artaxo, P., Brandão, C. *et al.* Biogeochemical cycling of carbon, water, energy, trace gases, and aerosols in Amazonia: the LBA-EUSTACH experiments. J. Geophys. Res. Atmos. **2002**, 107(D20), 8066.

Andreae, M.O., Rosenfeld, D. Aerosol‐cloud‐precipitation interactions. Part 1. The nature and sources of cloud‐active aerosols. *Earth-Sci. Rev.* **2008**, 89, 13–41.

Butler, B.W.; Cohen, J.; Latham, D.J.; Schuette, R.D.; Sopko, P.; Shannon, K.S., Jimenez, D.; Bradshaw, L.S. Measurements of radiant emissive power and temperatures in crown fires. *Can. J. For. Res.* **2004**, 34, 1577-1587.

Butler, B.; Teske, C.; Jimenez, D.; O’Brien, J.J.; Sopko, P.; Wold, C.; Vosburgh, M.; Hornsby, B.; Loudermilk, E. Observations of fire intensity and fire spread rate—RxCADRE 2012. *Int. J. Wildland Fire* **2016**, 25, 76-89.

Cheney, N.P.; Gould, J.S.; Catchpole, R.W. The influence of fuel, weather and fire shape 794 variables on fire spread in grasslands. *Int. J. Wildland Fire* **1993**, 3, 31–44.

Cheney, N.P.; Gould, J.S.; Catchpole, R.W. Prediction of fire spread in grasslands. *Int. J. Wildland Fire* **1998**, 9, 1–13.

Clements, C.B. Thermodynamic structure of a grass fire plume. *Int. J. Wildland Fire* **2010**, 19: 895-902.

Clements, C.B.; Zhong, S.; Bian, X.; Heilman, W.E.; Byun D.W. First observations of turbulence generated by grass fires, *J. Geophys. Res.* **2008**, 113, D22102.

Clements, C.B.; Kukkonen, J.; de Leeuw, G.; Virkkula, A.; Levula, J.; Schultz, D.M.; Kulmala, M. An overview of atmospheric measurements made during the IS4FIRES experiments at Hyytiälä, Finland. In *Proceedings of the* *Eighth Symposium on Fire and Forest Meteorology*, 12-15 October 2009, Kalispel, Montana, American Meteorological Society.

Clements, C.B.; Lareau, N.; Seto, N.; Contezac, J.; Davis, B.; Teske, C.; Butler, B.; Jimenez, D. Meteorological measurements and fire weather conditions-RxCADRE 2012. *Int. J. Wildland Fire* **2016**, 25, 90-101.

Clements, C.B.; Zhong, S.; Goodrick, S.; Li, J.; Bian, X.; Potter, B.E.; Heilman, W.E.; Charney, J.J.; Perna, R.; Jang, M.; Lee, D.; Patel, M.; Street, S.; Aumann, G. Observing the Dynamics of Wildland Grass Fires: FireFlux- A Field Validation Experiment. *B. Am. Meteorol. Soc.* **2007**, 88, 1369-1382.

deGroot, W.J.; Bothwell, P.M.; Taylor, S.W.; Wotton, B.M.; Stocks, B.J.; Alexander, M.E. Jack pine regeneration and crown fires. *Can. J. For. Res.* **2004**, 34, 1634-1641.

Delmas, R.A.; Druilhet, A.; Cros, B. *et al.* Experiment for Regional Sources and Sinks of Oxidants (Expresso): An Overview. *J. Geophys. Res. Atmos.* **1999**, 104, 30609-30624.

Dickinson, M.B.; Hudak, A.T.; Zajkowski, T.; Loudermilk, E.L.; Schroeder, W.; Ellison, L.; Kremens, R.L.; Holley, W.; Martinez, O.; Paxton, A.; Bright, B.C.; O’Brien, J.J.; Hornsby, B.; Ichoku, C.; Faulring, J.; Gerace, A.; Peterson, D.; Mauceri, J. Measuring radiant emissions from entire prescribed fires with ground, airborne and satellite sensors – RxCADRE 2012. Int. J. Wildland Fire **2016**, 25, 48-61.

Fingas, M.F. The Newfoundland Offshore Burn Experiment – NOBE. In Proceedings of the 1995 International Oil Spill Conference. American Petroleum Institute, Publication 4620. pp 123-132, **1996**.

FIRESCAN Science Team. Fire in ecosystems of boreal Eurasia: The Bor Forest Island Fire Experiment, Fire Research Campaign Asia-North (FIRESCAN). In: *Biomass burning and global change*. Vol. II (J.S. Levine, ed.), The MIT Press, Cambridge, MA, pp 848-873, 1996.

Keene, W.C.; Lobert, R.M.; Crutzen, P.J.; Brain, C. Emissions of major gaseous and particulate species during experimental burns of southern African biomass. *J. Geophys. Res.* **2006**, 111, D04301.

Goldammer, J.G. Consequences and implications: Synthesis of the STARE/SAFARI experiment, in van Wilgen, V.W.; Andreae, M.O.; Goldammer, J.G.; Lindesay, J.A. (eds.), Fire in the Southern African Savanna: Ecological and Environmental Perspectives, Witwatersrand University Press, Johannesburg, South Africa, pp. 239-249, **1997**.

Gould, J.S.; McCaw, W.L.; Cheney, N.P.; Ellis, P.F.; Knight, I.K.; Sullivan, A.L.Project Vesta: fire in dry eucalpyt forest. Ensis-CSIRO, Canberra ACT and Department of Environment and Conservation, Perth, Australia, **2008**.

Harden, J.W.; Neff, J.C.; Sandberg, D.V.; Turetsky, M.R.; Ottmar, R.; Gleixner, G.; Fries, T.L.; Manies, K.L. Chemistry of burning the forest floor during the FROSTFIRE experimental burn, interior Alaska, 1999. *Global Biogeochem. Cycles* **2004**, 18, GB3014.

Heilman, W.E.; Zhong, S.; Hom, J.L.; Charney, J.J. Development of modeling tools for predicting smoke dispersion from low-intensity fires. Joint Fire Science Program, **2013**, Final Report 09-1-04-1.

Hoffa EA, Ward DE, Hao WM, Susott RA, Wakimoto RH. Seasonality of carbon emissions from biomass burning in a Zambian savanna. *J. Geophys. Res. Atmospheres* **1999**, 104(D11), 13841-53.

Hudak, A.T.; Dickinson, M.B.; Bright, B.C.; Kremens, R.L.; Loudermilk, L.E.; O’Brien, J.J.; Hornsby, B.; Ottmar, R.D. Measurements relating fire radiative energy density and surface fuel consumption—RxCADRE 2011 and 2012. *Int. J. Wildland Fire* **2016**, 25, 25-37.

Jacob, D.J.; Crawford, J.H.; Maring, H.; Clarke, A.D.; Dibb, J.E.; Emmons, L.K.; Ferrare, R.A.; Hostetlerr, C.A.; Russell, P.B.; Singh, H.B.; Thompson, A.M.; Shaw, G.E.; McCauley, E.; Pederson, J.R.; Fisher, J.A. The Arctic research of the composition of the troposphere from aircraft and satellites (ARCTAS) mission: design, execution, and first results. *Atmos. Chem. Phys.* **2010**, 10, 5191-5212.

Keene, W.C.; Lobert, J.M.; Crutzen, P.J.; Maben, J.R.; Scharffe, D.H.; Landmann, T.; Hely, C.; Brain, C. Emissions of major gaseous and particulate species during experimental burns of southern African biomass. *J. Geophys. Res.* **2006**, 111, D04301.

Linn, R.; Anderson, K.; Winterkamp, J.; Brooks, A.; Wotton, M.; Dupuy, J.L.; Pimont, F.; Edminster, C. Incorporating field wind data into FIRETEC simulations of the International Crown Fire Modeling Experiment (ICFME): preliminary lessons learned. *Can. J. For. Res.* **2012**, 42, 879-898.

McGratton, K.B.; Baum, H.R.; Walton, W.D.; Trelles, J. *Smoke plume trajectory from in situ burning of crude oil in Alaska field experiments and modeling of complex terrain*. US Department of Commerce National Institute of Standards and Technology. **1995**, Report number 5958.

McGratton, K.B.; Baum, H.R.; Walton, W.D. Smoke plume trajectory from in situ burning of crude oil in Alaska: field experiments and modeling of complex terrain. National Institute of Structural Technology, **1998**, Report Number 5958.

O’Brien, J.; Loudermilk, L.E.; Hornsby, B.; Hudak, A.T.; Bright, B.C.; Dickinson, M.B.; Hiers, K.J.; Ottmar, R.D. High resolution infrared thermography measurements for capturing fire behavior in wildland fires—RxCADRE 2012. *Int. J. Wildland Fire* **2016**, 25, 62-75.

Ottmar, R.D.; Hiers, K.H.; Clements, C.B.; Butler, B.; Dickinson, M.B.; Potter, B.; O’Brien, J.J.; Hudak, A.T.; Rowell, E.M.; Zajkowski, T.J. Measurements, datasets and preliminary result from the RxCADRE project-2008, 2011 and 2012. *Int. J. Wildland Fire* **2016**, *2*5, *1-9.*

Ottmar, R.D.; Hudak, A.T.; Wright, C.S.; Vihnanek, R.E.; Restaino, J.C. Pre- and post-fire surface fuel and cover measurements—RxCADRE 2008, 2011, and 2012. *Int. J. Wildland Fire* **2016**, 25, 10-24.

Pereira, G.; Siqueira, R.; Rosário, N.E.; Longo, K.L.; Freitas, S.R.; Cardozo, F.S.; Kaiser, J.W.; Wooster, M.J. Assessment of fire emissions inventories during the South American biomass burning analysis (SAMBBA) experiment. *Atmos. Chem. Phys. Discuss.* **2016**, Jan 19:1-23.

Putnam, T.; Butler, B.W. Evaluating fire shelter performance in experimental crown fires. *Can. J. For. Res.* **2004**, 34, 1600-15.

Ross, J.L.; Ferek, R.; Hobbes, P.V. Particle and gas emissions from an in situ burn of crude oil on the ocean. *J Air Waste Manage. Assoc.* **1996**, 46, 251-259.

Rowell, E.M.; Seielstad, C.A.; Ottmar, R.D. Development and validation of fuel height models for terrestrial lidar - RxCADRE 2012. *Int. J. Wildland Fire* **2016**, 25, 38-47.

Strand, T.; Gullett, B.; S. Urbanski, S.O. O’Neill, B. Potter, J. Aurell, A. Holder, N. Larkin, M. Moore, M. Rorig. Grassland and forest understorey biomass emissions from prescribed fires in the southeastern United States – RxCADRE 2012. *Int. J. Wildland Fire* **2016***,* 25, 102-113.

Stocks, B.J.; Alexander, M.E.; Lanoville, R.A. Overview of the International Crown Fire Modeling Experiment (ICFME). *Can. J. For. Res.* **2004a**, 34, 1543-1547.

Stocks, B.J.; Alexander, M.E.; Wotton, B.M.; Stefner, C.N.; Flannigan, M.D.; Taylor, S.W.; Lavoie, N.; Mason, J.A.; Hartley, G.R.; Maffey, M.E.; Dairymple, G.N.; Blake, T.W.; Cruz, M.G.; Lanoville, R.A. Crown fire behavior in a northern jack pine – black spruce forest. *Can. J. For. Res.* **2004b**, 34, 1548-1560.

Stocks, B.J. Black spruce crown fuel weights in northern Ontario. *Can. J. For. Res.* **1980**, 10, 498-501.

Stocks, B.J. Fire behavior in immature jack pine. *Can. J. For. Res.* **1987a**, 17, 80-86.

Stocks, B.J. Fire potential in the spruce budworm-damaged forests of Ontario. *Forest. Chron.* **1987b**, 63, 8-14.

Stocks, B.J. Fire behavior in mature jack pine. *Can. J. For. Res.* **1998**, 19, 783-790.

Strand, T.; Rorig, M.; Yedinak, K.; Seto, D.; Allwin, E.; Garci, F.A.; O’Keef, P.O.; Checans, V.C.; Mickler, R.; Clements, C.; Lamb, B. Subcanopy transport and dispersion of smoke. Final Report to the Joint Fire Science Program, **2013**, Final Report 09-1-04-2.

Tsay, S.C.; Hsu, C.; Lau, W.K.M. *et al.* From BASE-ASIA toward 7-SEAS: A satellite-surface perspective of boreal spring biomass-burning aerosols and clouds in Southeast Asia. *Atmos. Environ.* **2013**, 78, 20-34.

Walton, W.D.; Twiley, W.H.; Putorti, A.D.; Hiltabrand, R.R. Smoke measurements using an advanced helicopter transported sampling package with radio telemetry. In *Proceedings of the 18^th^ Arctic and Marine Oil Spill Program (AMOP) Technical Seminar*. Environment Canada Emergencies Science Division, Ottawa, Canada. pp 1053-1074, **1995**.

Wang, S.H.; Tsay, S.C.; Lin, N.H.; Hsu, N.C.; Bell, S.W.; Li, C.; Ji, Q.; Jeong, M.J.; Hansell, R.A.; Welton, E.J.; Holben, B.N. First detailed observations of long-range transported dust over the northern South China Sea. *Atmos. Environ.* **2011**, 45, 4804-4808.

Wang, S.H.; Tsay, S.C.; Lin, N.H.; Chang, S.C.; Li, C.; Welton, E.J.; Holben, B.N.; Hsu, N.C.; Lau, W.K.; Lolli, S.; Kuo, C.C. Origin, transport, and vertical distribution of atmospheric pollutants over the northern South China Sea during the 7-SEAS/Dongsha Experiment. *Atmos. Environ.* **2013**, 78, 124-33.

Warneke, C.; Schwarz, J.P.; Ryerson, T.; Crawford, J.; Dibb, J.; Lefer, B.; Roberts, J.; Trainer, M.; Murphy, D.; Brown, S.; Brewer, A.; Gao, R.-S.; Fahey, D. Fire influence on regional global environments and air quality (FIREX-AQ): a NOAA/NASA interagency intensive study of North American Fires, **2018**. https://www.esrl.noaa.gov/csd/projects/firex/whitepaper.pdf

Williams, R.J.; Gill, A.M.; Moore, P.H.R. Seasonal changes in fire behavior in northern Australia. *Int. J. Wildland Fire* **1998**, 8, 227-239.
